# Injection and controlled motion of conducting domain walls in improper ferroelectric Cu-Cl boracite

**DOI:** 10.1038/ncomms15105

**Published:** 2017-05-16

**Authors:** Raymond G.P. McQuaid, Michael P. Campbell, Roger W. Whatmore, Amit Kumar, J. Marty Gregg

**Affiliations:** 1 Centre for Nanostructured Media, School of Mathematics and Physics, Queen's University Belfast, Belfast BT7 1NN, UK; 2 Department of Materials, Imperial College London, Exhibition Road, London SW7 2AZ, UK

## Abstract

Ferroelectric domain walls constitute a completely new class of sheet-like functional material. Moreover, since domain walls are generally writable, erasable and mobile, they could be useful in functionally agile devices: for example, creating and moving conducting walls could make or break electrical connections in new forms of reconfigurable nanocircuitry. However, significant challenges exist: site-specific injection and annihilation of planar walls, which show robust conductivity, has not been easy to achieve. Here, we report the observation, mechanical writing and controlled movement of charged conducting domain walls in the improper-ferroelectric Cu_3_B_7_O_13_Cl. Walls are straight, tens of microns long and exist as a consequence of elastic compatibility conditions between specific domain pairs. We show that site-specific injection of conducting walls of up to hundreds of microns in length can be achieved through locally applied point-stress and, once created, that they can be moved and repositioned using applied electric fields.

## Abstract (web summary)

Conducting ferroelectric domain walls constitute a new class of functional material, but to achieve site-specific injection and annihilation of such walls is challenging. Here, McQuaid *et al*. report site-specific injection of such walls in Cu_3_B_7_O_13_Cl created by local point-stress and controlled by electric field.

Identifying and understanding the key factors that generate reliable, well-controlled conductivity along ferroic domain walls is a crucial step towards realizing prototype domain wall-based nanoelectronic devices^1^,^2^,^3^,^4^,^5^,^6^. In the original landmark observations of domain wall conductivity in thin-films of BiFeO_3_ (ref. 5) significant densities of charge-carrying point defects were seen to play a decisive role in determining wall transport^6^. This situation occurs readily in naturally leaky systems and can be created artificially by either extrinsic doping^6^,^7^, or by vacuum annealing, as demonstrated in thin-films of ferroelectric Pb(Zr,Ti)O_3_ (ref. 8). This offers a large degree of flexibility in engineering domain wall transport but without strict defect control or quantification can lead to difficulties with consistency and reproducibility in conduction, such as is seen to be the case in 71° walls in BiFeO_3_ (ref. 9).

The engineering of polar discontinuities across domain walls is an attractive alternative to the extrinsic manipulation of domain wall transport through sample processing. Conductivity anomalies have been rationalized as an intrinsic feature of these charged-type domain walls, where the near-field dipolar configuration either side of the boundary is oriented head-to-head or tail-to-tail and orders of magnitude enhancement of local conductivity along charged domain walls have been reported^10^,^11^,^12^. However, in proper ferroelectrics, where polarization is the primary order parameter, such polar discontinuities usually are highly unstable and spontaneously forming charged-wall configurations are therefore rarely seen to occur^13^. The situation can be very different in improper ferroelectrics. These are materials where the primary order parameter is not polarization but instead is a physical quantity, such as spontaneous strain, which exhibits different transformation properties^14^. A crucial aspect of the classification is that the development of polarization is unable to fully account for the reduction in symmetry through the transition (and therefore cannot be the primary order parameter). The rare-earth manganites provide a textbook case, where development of the primary order parameter upon cooling through the transition, comprising of a structural tilting of MnO_5_ trigonal bipyramids (known as trimerization)^15^, also leads to a spontaneous polarization developing as a secondary order parameter. The mechanical compatibility of structural domain variants, which requires matching of the domain-related deformations at the boundary plane, as formalized by Fousek and Janovec^16^, is seen to take precedence over electrostatic near-field neutrality and enables proliferation of stable charged conducting domain walls^2^. A somewhat analogous case has been reported more recently with the observation of charged walls in improper ferroelectric-layered-perovskite (Ca,Sr)_3_Ti_2_O_7_ (ref. 11). Here, rotations/tilting of TiO_6_ octahedra leads to a ferroelastic domain structure but also the concurrent development of a spontaneous polarization, due to an accompanying displacement of the Ca/Sr ions. Like in manganites, mechanical compatibility between structural domain variants takes precedence over polar continuity, enabling spontaneous formation of charged type domain walls. However, the existence of improper ferroelectricity is not a sufficient criterion to ensure charged-wall formation. For example, in ferroelastic improper-ferroelectric Gd_2_(MoO_4_)_3_, which undergoes a transition from paraelectric 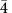
2*m* to ferroelectric/ferroelastic *mm*2 (ref. 17), only uncharged 180° walls are expected to be stable^18^. To determine the charge or neutral state of the walls, one must examine the expected equilibrium domain wall configurations associated with the primary order parameter and identify the possible polar configurations at the wall, based on the explicit orientation relationship between the primary order parameter and polarization. Using the approach pioneered by Fousek and Janovec^16^, Erhart has carried out a comprehensive survey of permissible domain wall orientations for all ferroelastic/ferroelectric species based on mechanical compatibility considerations^18^ and lists cases where charged walls may be expected.

As it stands, improper ferroelectrics are promising candidates for exhibiting conducting domain walls with robust transport characteristics but so far they have not been successfully used to deliver site-specific wall deployment and positioning. For example, in the manganites the fact that the walls are curved and consist of irregular sections with enhanced transport makes it difficult to envisage how they can be easily manipulated in a device. As-grown improper-ferroelectric (Ca,Sr)_3_Ti_2_O_7_ crystals^11^ have the advantage of exhibiting planar rectilinear conducting walls, but a means for their controlled injection and positioning has not been shown. Indeed, there is no evidence at present to suggest that they persist after thermal or electric field cycling. In contrast, techniques for controlling domain walls are better-established for conventional proper ferroelectrics. Local writing of conducting walls using a biased atomic force microscope probe has proved effective and popular for thin-films^3^,^5^,^8^,^12^. Even so, more device-relevant designs, such as that explored by Sluka and co-workers at EPFL^10^,^19^ in bulk BaTiO_3_ crystals, lack site-specific injection and control capabilities.

In this article we report site-specific injection and positional control of conducting charged domain walls in proper-ferroelastic improper-ferroelectric Cu_3_B_7_O_13_Cl single crystals. In this system, mechanical compatibility conditions between specific domain pairs can lead to generation of a 90° head–head/tail–tail polar configuration. Spontaneously formed charged boundaries are seen to be rectilinear and of order tens of microns in length. We show that local mechanical stress can be used for repeatable site-specific injection of conducting wall arrays that span up to hundreds of microns in length and then show how individual walls can be moved using applied planar electric fields. Such an approach may be useful in the design of piezoresistive-type devices^20^, where functionality is derived from stress-induced conducting domain walls.

## Results

### Boracite crystal structure

**Figure 1:**
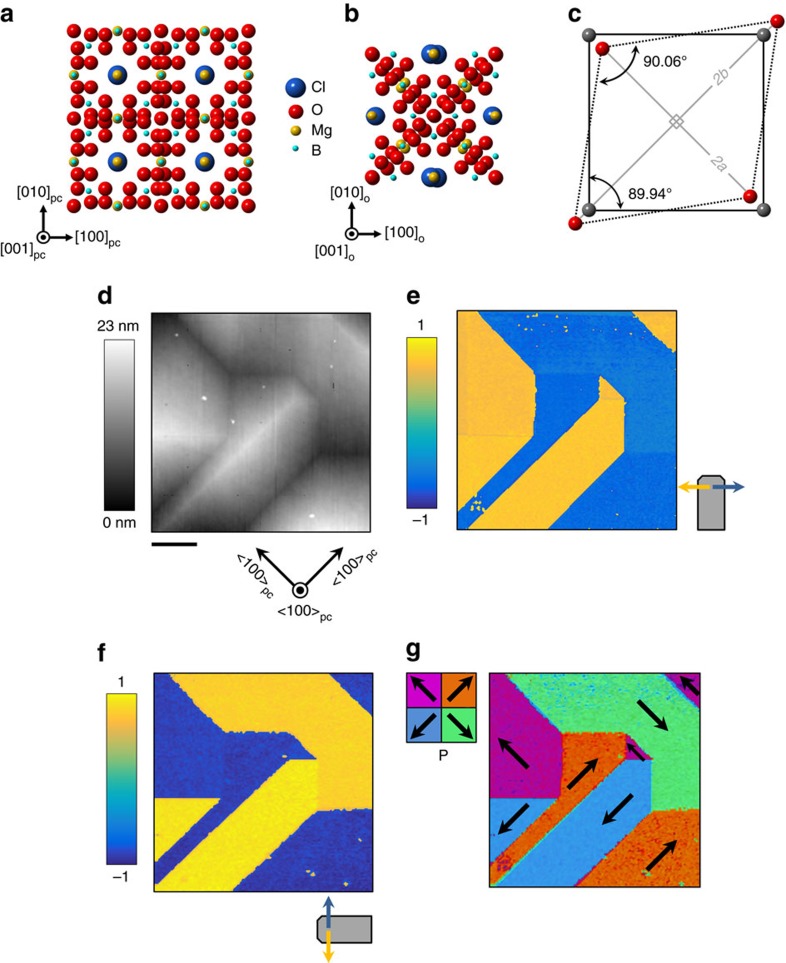
Crystal structure and domains in boracites. Atomic structure of the Mg_3_B_7_O_13_Cl unit cell projected on (001)_cubic_ for the cubic phase (**a**) and (001)_pseudocubic_ for the orthorhombic phase (**b**). (**c**) The shear strain associated with the transition is revealed by showing the relative position of oxygen atoms common to four unit cells in the cubic phase (schematized in grey) and for the orthorhombic phase (in red). The shear distortion is exaggerated by a factor of 300. Labels *a* and *b* correspond to the orthorhombic lattice parameters. Labelled angles correspond to the true values expected for the strained unit cell for Mg–Cl boracite. (**d**) Local topographical map of Cu–Cl boracite crystal surface as measured by atomic force microscopy. The scale bar measures 2 μm. (**e**) Corrected lateral-mode piezoresponse map (a.u.) for the area shown in **d** and scan obtained with relative cantilever orientation at 90°, (**f**). Grey motif indicates cantilever orientation. (**g**) Montage of piezoreponse maps in **e**,**f**. Coloured legend represents the four in-plane polarized domain states with orientations labelled by arrows.

The family of minerals commonly described as boracites have composition M_3_B_7_O_13_X, where M is a divalent metal and X is usually a halogen (but can also be OH^−^ or S^2−^ (ref. 21)). Boracites have a complex crystal structure with a large unit cell^22^ and undergo a ferroelastic transition from cubic point group 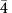
3*m* to orthorhombic *mm2* (ref. 23). The natural boracite (Mg_3_B_7_O_13_Cl) mineral structure can be envisaged as a three dimensional unbroken B–O network with Mg^2+^ and Cl^−^ ions filling the interstices, as illustrated in Fig. 1a,b (further details in Supplementary Fig. 1). The B–O network consists of corner-sharing BO_4_ tetrahedra and BO_3_ triangles with each Mg^2+^ ion being octahedrally coordinated with four oxygens and two chlorines. Upon cooling through the transition, a structural shearing of the cubic unit cell occurs^24^ (Fig. 1c). The transition is improper ferroelectric in character: alternate layers of Cl^−^ ions shift along different 〈111〉_pseudocubic (pc)_ directions leading to a net displacement of the centre of negative charge along [001]_pc_ while all Mg^2+^ ions shift along 〈100〉_pc_ directions^25^. For the Mg^2+^ ions, two perpendicular sublattices can be identified where the ions perform antiparallel shifts and a third where the net displacement is along [00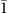]_pc_ (ref. 25). The net relative shifts of the halogen and metal ions along [001]_pc_ lead to the development of a dipole moment along this axis (and the possibility for six equivalent 〈100〉_pc_ oriented polar variants upon symmetry breaking from the high-temperature cubic state).

### Identification of charged walls and conductivity mapping

Local surface topography of a {100}_pc_-faced Cu–Cl boracite single crystal plate, measured using atomic force microscopy (AFM; Methods section), is shown in Fig. 1d. The observed surface deformations are consistent with the development of ferroelastic shear twins having boundaries with surface trace along 〈100〉_pc_ and 〈110〉_pc_ (ref. 26). Lateral-mode piezoresponse force microscopy (PFM; Methods section) of the same area in Fig. 1e–g sheds light on the polar domain structure; there is clearly spatial variation in the measured piezoresponse signals that correlates with the spatial distribution of ferroelastic domains. The piezoresponse maps presented in Fig. 1e,f have been corrected to remove a deflection-related cross-talk signal (due to low signal-to-noise associated with the genuine piezoresponse, see Supplementary Note 1). Combining the information in Fig. 1e,f into a single-domain map, the polarization of each domain can be assigned as shown in Fig. 1g. All the topographical boundaries from Fig. 1d can be identified in this image, indicating that the surface topography delineates the polar domain structure as well as the ferroelastic domains (as expected in boracites^24^). Vertical PFM maps show poor signal-to-noise with measured contrast that depends on sample orientation, suggesting that the signal is either flexural in origin and/or heavily influenced by deflection-related crosstalk. We therefore expect that all polarization lies in the plane parallel to the sample surface.

**Figure 2:**
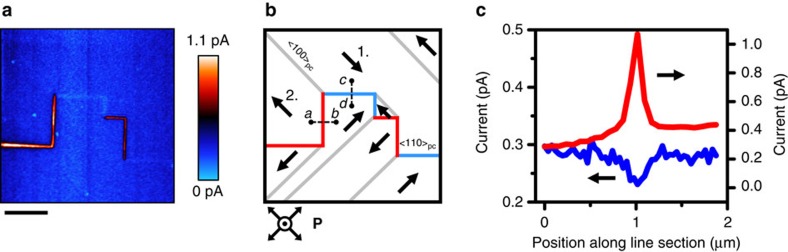
Transport along charged walls. (**a**) Spatially resolved current map captured with −12 V_dc_ applied to the bottom electrode. The bulk background signal (order of 0.1 pA) has been removed to enhance wall-specific contrast. The scale bar measures 2 μm. (**b**) Schematic of polar domain structure with 90° head-to-head and tail-to-tail charged boundaries coloured in blue and red respectively. Uncharged 180° walls are indicated as grey lines. (**c**) Current line-sections taken across the conducting and insulating walls seen in **a**. The conducting wall line profile (red) is taken across the dashed line path *a* to *b* in **b** while the insulating wall profile is taken along the path *c* to *d*.

Nanoscale spatially-resolved current mapping (described in Methods section) is shown in Fig. 2a for the polydomain region imaged in Fig. 1d–g. Specific ferroelectric/ferroelastic domain walls (identified in Fig. 2b schematic) are seen to exhibit enhanced conductivity compared to the bulk. Also visible, but much fainter in signal, are boundaries that appear more resistive than the bulk. These measurements support Schmid and Pétermann's early speculation^27^, based on indirect bulk conductivity measurements, that domain walls in single crystal Cu–Cl boracite may be conducting. Using the PFM analysis of Fig. 1, we can examine the polar structure, schematized in Fig. 2b, allowing us to identify head-to-head 90° domain walls, tail-to-tail 90° walls and uncharged 180° domain walls. Schematic illustrations of how the sheared unit cell leads to the observed domain wall orientations and characteristic surface distortions are shown in Supplementary Figs 5 and 6. Comparison with the current map in Fig. 2a immediately reveals a direct correlation between the predicted charge-state of each wall and the local current amplitude experimentally measured. Tail–tail charged 90° walls are seen to be solely responsible for the enhanced conduction and head–head charged 90° walls account for the insulating walls. The observation that uncharged 180° domain walls exhibit no current anomaly is further strong evidence that it is the charge state of the boundary that underpins the augmented or diminished local transport. The spontaneous development of charged walls in boracites is not surprising, as Schmid and co-workers have previously determined that the only mechanically compatible ferroelastic domain walls with composition plane {110}_pc_ correspond to the case of 90° charged walls, as seen in our experiments^24^,^25^. Knowing this, we can determine the domain polarization map (to within a 180° rotation of all polarization vectors) using solely the sheared domain topography as measured by atomic force microscopy (see Supplementary Note 2). We note that observation of conductivity enhancement/suppression being directly correlated with predicted charged-wall species is entirely consistent with local domain wall transport measurements made in other improper-ferroelectric crystals to date (ErMnO_3_ (ref. 2) and in (Ca,Sr)_3_Ti_2_O_7_ (ref. 11)) as well as in proper ferroelectric BaTiO_3_ (ref. 10). We suggest that conduction in boracites is mediated by *p*-type majority carriers based on bulk Hall effect measurements (see Supplementary Note 3). The observation of tail–tail conducting walls here is therefore consistent with the prevailing theory that enhanced conductivity is observed along walls that are screened by majority-type carriers^2^,^28^.

### Pressure writing of charged domain walls

**Figure 3:**
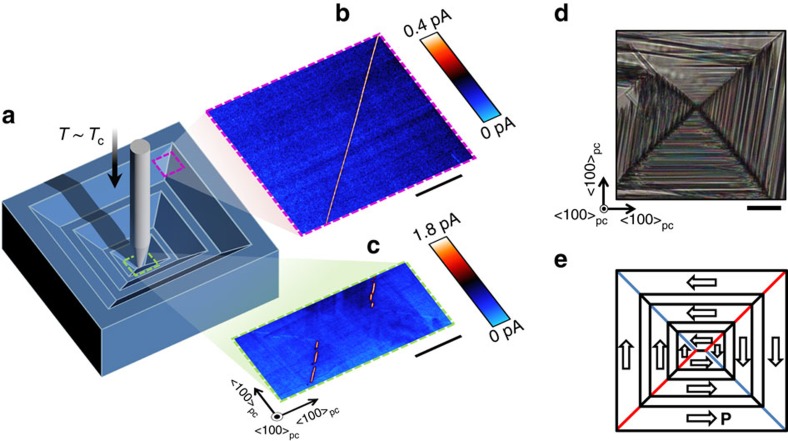
Stress-field injection of charged domain walls near the Curie temperature. (**a**) Schematic quadrant domain structure after probe-applied stress at elevated temperature. Spatially resolved current map of an injected long conducting boundary, (**b**), and the distribution of conducting and insulating boundaries surrounding the point where stress is applied, (**c**). A bias of magnitude −12 V_dc_ is applied to the bottom electrode. Differences in measured current values between panels (**b**,**c**) are due to wear of the conductive tip-coating rather than differences in the intrinsic transport properties of the boundary. The scale bars in (**b**,**c**) measure 10 μm. (**d**) Polarized light microscopy of the quadrant domain microstructure that develops around the point where probe-pressure is applied. (**e**) Schematic illustration of domain structure in **d** with in-plane domain polarizations indicated. Insulating head-to-head charged 90° walls are indicated with blue lines and conducting tail-to-tail 90° charged walls are indicated with red lines. Uncharged boundaries are indicated with black lines. The scale bar measures 100 μm.

We also demonstrate a technique for creating ordered patterns of these conducting domain walls using mechanical pressure, shown in Fig. 3. First, the crystal is heated to one degree below the phase transition (*T*_c_=91 °C (ref. 29)) such that domain wall mobility is increased compared to room temperature and ferroelastic transformation plasticity developed. Next, a fine-tipped metal probe is pressed into the surface of the crystal. With applied pressure of order 1 GPa, dramatic domain reconfiguration is observed to occur around the contact point, up to hundreds of microns away (Fig. 3d), similar to that seen in nanoindentation experiments carried out on ferroelectrics^30^. The temperature is then reduced and the surface stress removed, leaving a depression formed by quadrants consisting of finer-scale domains. The pressure required to write this pattern is larger than the uniaxial pressure of 120 MPa reported by Torre *et al*.^31^ required for room temperature ferroelastic domain switching in Mg–Cl boracite; we expect our 1 GPa estimate to be an upper limit since the magnitude of applied stress will decrease away from the point of contact (as opposed to uniform stress applied in the study by Torre *et al*.^31^). Current mapping (Fig. 3b,c) shows that two of the written quadrant boundaries consist of conducting domain wall sections that extend in the same direction for hundreds of microns in length (Fig. 3d). Artificially written conducting walls can be over 50 μm in length, as shown in Fig. 3b, and on occasion can be hundreds of microns long. The boundaries have a surface trace along 〈110〉_pc_ indicating that the observed conductivity enhancement is consistent with the 90° charged-wall interpretation described above. The other two opposing quadrant boundaries that trace along 〈110〉_pc_ directions are therefore expected to have an in-plane polar discontinuity of the opposite charge sense (made clear in the schematic in Fig. 3e). Indeed, close examination of the current profile reveals segments that are more resistive than the bulk (Fig. 3c), consistent with the charged-wall picture. From a thermodynamics perspective, the specific quadrant pattern of ferroelastic domains can be rationalized to occur since they facilitate a depression of the surface in the immediate vicinity of the applied pressure. The strict orientational relationship between the structural shearing and polarization allows us to identify that domains which generate surface corrugation have polarization that is restricted to being in-plane and oriented perpendicular to the shear gradient. These mechanically written conducting wall patterns appear stable, showing no discernible change after almost 2 weeks and the writing process is seen to cause no damage in the vicinity of the probe-contact.

An important feature of this quadrant pattern with conducting domain walls along {110}_pc_ is that it can be formed reliably and site-specifically when this heat-assisted stress poling procedure is repeated. The pattern can be then erased by thermal-cycling through the modest phase transition temperature, annihilating the conducting walls. Using the approach pioneered by Gruverman and co-workers^32^, basic prototype devices that are actuated by nanoprobe-induced stress may be a possibility if boracite thin-films can be fabricated (to date, only Schmid *et al*.^33^ have attempted film growth). Some degree of scope for tuning the transport properties of the boracite system may be anticipated by appropriate choice of the metal–halogen combination. It should also be possible to write these charged domain wall patterns with point-like stress in other 〈100〉_pc_-faced orthorhombic boracites since they should exhibit the same geometric shear response and therefore the same charged polar configuration. In fact, a strikingly similar charged-wall configuration and quadrant microstructure has even been seen to form naturally in Mg_3_B_7_O_13_Cl boracite crystals due to environmental stresses associated with their petrogenesis^25^,^34^.

### Electric field control of charged domain wall position

**Figure 4:**
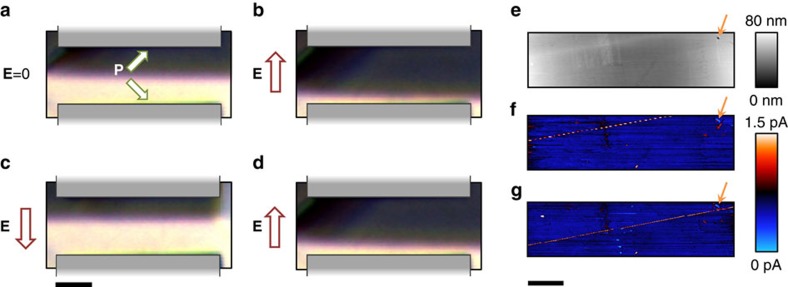
Electric field control of conducting domain walls near the Curie temperature. (**a**) Polarized light microscopy of a conducting domain wall under zero applied field (**E**). (**b**) **E**-field applied between surface planar electrodes (schematized in grey) in positive sense causes wall to move towards bottom electrode. Arrows labelled **P** denote domain polarization direction. (**c**) Reversing applied field direction causes the wall to move towards the top electrode. (**d**) Field applied again in positive direction causes wall to move back towards the bottom electrode. The scale bar for **a**–**d** measures 25 μm. Topography, (**e**) and spatially resolved current map (**f**) of the conducting wall shown in **a** before switching fields are applied. (**g**) Spatially resolved current map of the conducting wall after switching shows that its transport properties are not changed by wall movement. The orange arrow points to a topographical marker which serves as a reference for relative wall position. The apparent ‘beaded' profile of the conducting wall is an imaging artefact due to the cantilever scan axis being at an oblique angle to the wall. For nanoscale current imaging, a bias of −10 V_dc_ is applied to the bottom electrode. The scale bar for **e**–**g** measures 10 μm.

We are also able to experimentally demonstrate that these stress-written walls can be moved using applied electric fields, as shown in Fig. 4a–d. Gold planar electrodes were deposited on the surface and the sample was heated to 90 °C to increase charged-wall mobility. Using polarized light microscopy, we observed wall motion for electric field application of 30 kVcm^−1^ and above (electric field-oriented in-plane and perpendicular to the wall). Reversing the electric field caused the charged wall to move in the opposite direction, demonstrating that the position of these walls can be well controlled using electric field manipulation, after being created using applied stress. Crucially for applications, local current mapping (Fig. 4e–g), carried out both before and after switching, shows that moving the charged wall with electric field does not adversely affect its transport properties.

## Discussion

In summary, we have reported the first direct observation, controlled injection and field-controlled movement of conducting domain wall patterns in Cu–Cl boracite. Such domain walls are straight, can be tens or even hundreds of microns long and the observed conduction variations correlate directly with the charge state of the wall. We have demonstrated a simple site-specific domain wall writing technique using locally applied stress to reliably trigger growth of conducting domain wall patterns that span up to hundreds of microns in length. Furthermore, these walls can be repositioned using applied electric fields, an important requirement for conventional voltage-operated devices. For future work, parallel studies should be carried out on thin-film thicknesses of boracite material with highly localized pressure exerted using an atomic force microscope probe (such as in ref. 35). In particular, the density of domain walls injected using a local probe should be mapped fully as a function of temperature and pressure to explore the parameter space for which these conducting walls are stabilized.

## Methods

### Sample preparation and atomistic structure models

Single crystals of Cu_3_B_7_O_13_Cl, several millimetres in size, have been grown using a sealed ampoule, vapour phase transport technique^36^. Approximately 0.5 mm thick (100)_pc_-faced single crystals plates were obtained from the parent crystal by diamond sawing followed by a chemical-mechanical polish process. Cu–Cl boracite single crystals have a reported spontaneous polarization of ∼1.8 μC cm^−2^ and a coercive-field of up to 50 kV cm^−1^ as measured by Schmid *et al*.^37^. CrystalMaker software was used to make the atomistic models shown in Fig. 1 with atomic coordinates for Mg–Cl boracite as an input from the study by Ito *et al*.^22^.

### Scanning probe microscopy characterization

AFM topography mapping is carried out using a Veeco Dimension 3100 AFM system with a Nanoscope IIIa controller. PFM measurements are carried out using the same AFM system in conjunction with an EG&G 7265 lock-in amplifier. Commercially obtained Pt/Ir-coated Si probes are used (Nanosensors model PPP-EFM) with a probing signal of 5 V_ac_ at 20 kHz. Spatially resolved current mapping is carried out at room temperature using the AFM system in conjunction with a Bruker Tunnelling AFM module. Voltages up to −12 V_dc_ are applied to the base of the crystal while the tip is grounded.

### Electric field-driven motion of charged domain walls

For electric field-switching experiments, 100 nm of Au is sputter-deposited through a patterned transmission electron microscopy grid on top of the stress-written conducting domain wall pattern resulting in an array of 100 × 100 μm^2^ square-shaped surface electrodes. The crystal is then suspended across a hole in the centre of a ceramic heating element to allow for simultaneous heating (with temperature monitored by a thermocouple connected to a Thorlabs Model TC200 Controller) while transmission optical microscopy was carried out. A pair of electrodes located either side of the conducting wall are biased up to ±200 V_dc_ using a Keithley Model 237 source/measure unit and the charged domain wall position is then recorded using polarized light microscopy.

### Data availability

All data used to support the findings of this study are available from the corresponding author upon request.

## Additional information

**How to cite this article:** McQuaid, R. G. P. *et al*. Injection and controlled motion of conducting domain walls in improper ferroelectric Cu-Cl boracite. *Nat. Commun.* **8,** 15105 doi: 10.1038/ncomms15105 (2017).

**Publisher's note**: Springer Nature remains neutral with regard to jurisdictional claims in published maps and institutional affiliations.

## Supplementary Material

Supplementary Information Supplementary Figures, Supplementary Notes and Supplementary References
