# Supplementary material for: Injection and controlled motion of conducting domain walls in improper ferroelectric Cu-Cl boracite
Source: Nat Commun. 2017 May 16;8:15105. doi: 10.1038/ncomms15105 (PMC5440803; doi:10.1038/ncomms15105)
Supplement: Supplementary Information — Supplementary Figures, Supplementary Notes and Supplementary References [file ncomms15105-s1.pdf]

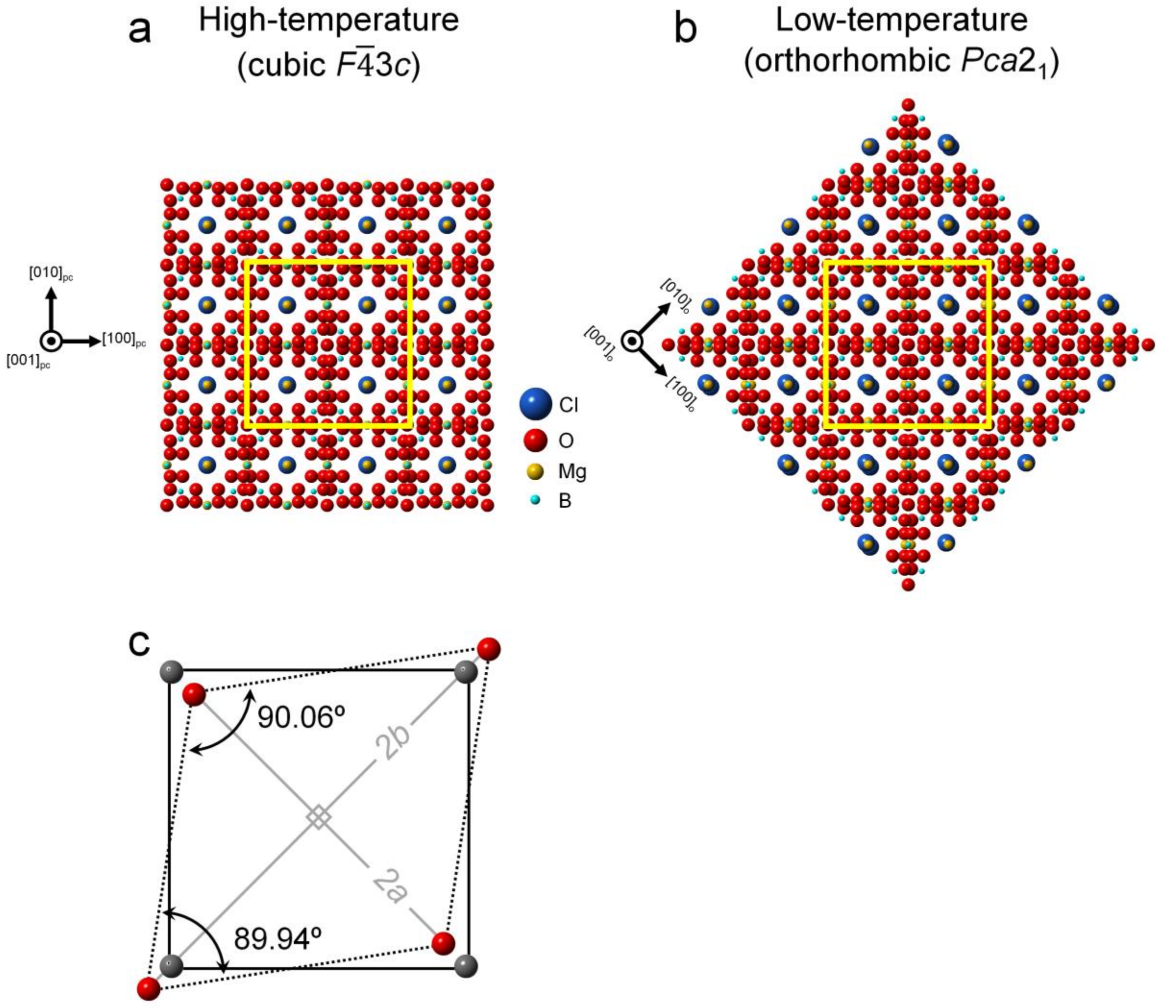

Supplementary Figure 1. Crystal structure of Mg-Cl boracite. (a) Cubic-phase atomic structure of a 2x2x1 $Mg_3B_7O_{13}Cl$ supercell projected on $(001)_{\text{cubic (c)}}$ for the cubic phase (space group $F\bar{4}3c$). (b) Orthorhombic-phase (space group $Pca2_1$) atomic structure of 3x3x1 supercell projected onto $(001)_{\text{orthorhombic (o)}}$. (c) The position of the oxygen atoms at the corners of the yellow squares in (a) and (b) are overlaid with the relative atomic displacements increased by a factor 300. Oxygen atoms in the cubic phase are schematised in grey and in red for the orthorhombic phase. Labels *a* and *b* correspond to the orthorhombic lattice parameters. Labelled angles correspond to the true values expected for the strained unit cell for Mg-Cl boracite. The models are created using CrystalMaker™ software with Ito *et al*.'s [1] atomic coordinate information for $Mg_3B_7O_{13}Cl$ as input. Upon cooling through the transition, the boron-oxygen network remains approximately unchanged but the metal/halogen coordination becomes altered with a given magnesium ion being closer to one halogen than the other (rather than symmetrically distributed as in the cubic phase). Detailed descriptions of the boracite structure can be found in references [1] and [2]. In Mg-Cl boracite the orthorhombic axes are defined as $a_o \cong 0.5\sqrt{2}\,a_c$, $b_o \cong 0.5\sqrt{2}\,a_c$, $c_o \cong a_c$, with a_o = 8.54169 Å, b_o = 8.55049 Å, and c_o = 12.09774 Å [3]. In Cu-Cl boracite the shear strain that develops ($[a_o\text{-}b_o]/[a_o+b_o]$) is more than four times larger than that in Mg-Cl boracite (~0.24 % compared to ~0.05 %).

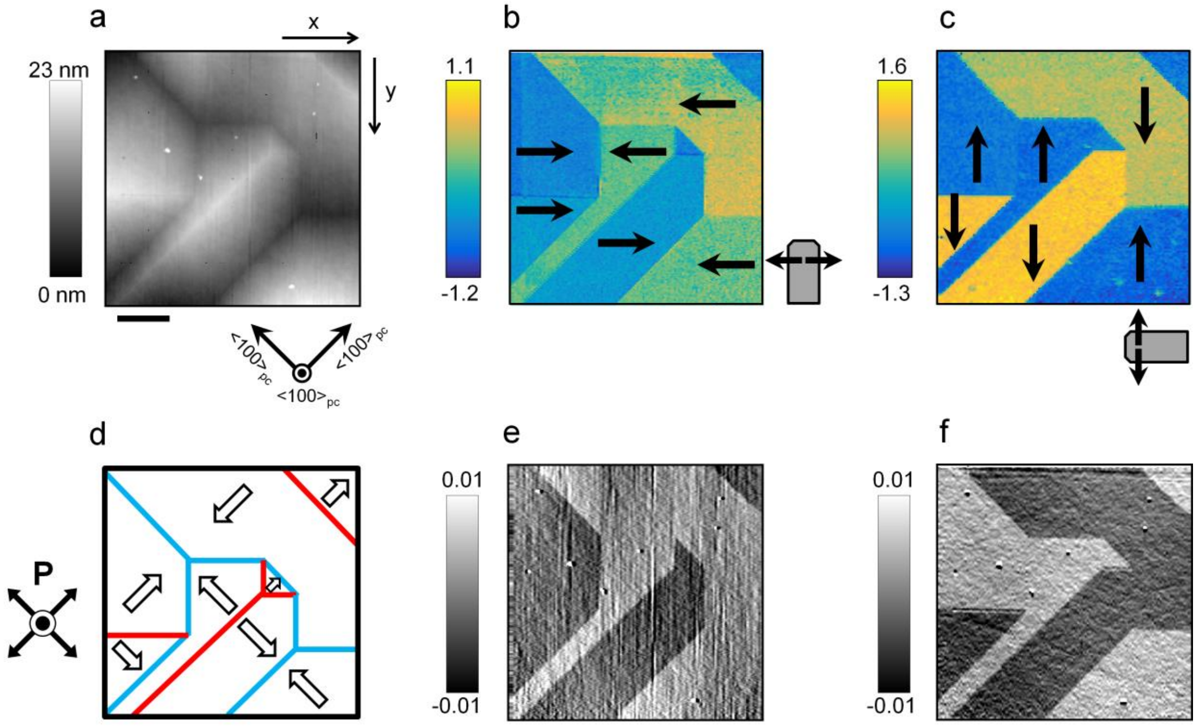

Supplementary Figure 2. Topography and piezoresponse force microscopy of Cu-Cl boracite. Topography map, (a), corresponding lateral piezoresponse map, (b), and lateral piezoresponse map obtained with sample rotated 90˚ with respect to the cantilever axis, (c). 5 V_{ac} was used for domain imaging. Black arrows denote direction of sensed polarisation component. (d) Calculated orientation of the polarisation (**P**) in the respective domains using as-measured piezoresponse maps. Predicted head-head and tail-tail charged walls are indicated in blue and red respectively. However, the prediction using as-measured data is invalid since it includes 180º charged domain walls which are not expected to be observed in this system (only 90º charged walls can form spontaneously in boracites). (e) Topography derivative $\partial z/\partial x$ and (f), $\partial z/\partial y$. Note the sign of derivative maps can be reversed depending on whether forward/reverse scan path is taken. The scale bar measures 2 μm.

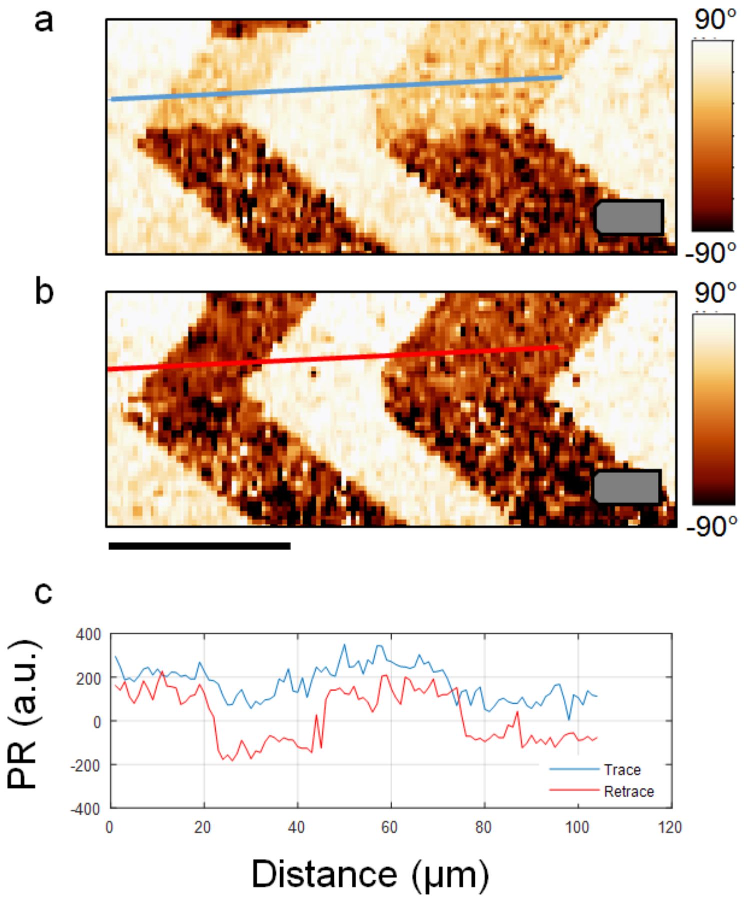

Supplementary Figure 3. Dependence of piezoresponse signal on scan path. (a) Trace (forward) and, (b), retrace (backward) scans of piezoresponse phase are shown for Cu-Cl boracite, captured using the same precise orientation of cantilever with respect to sample. (c) Comparing the piezoresponse (PR) along selected trace/retrace lines shows an unexpected inversion of phase. This shows that a deflection-related contribution to overall PFM signal can be significant enough to alter conventional domain map interpretation in this sample. The scale bar measures 5 μm.

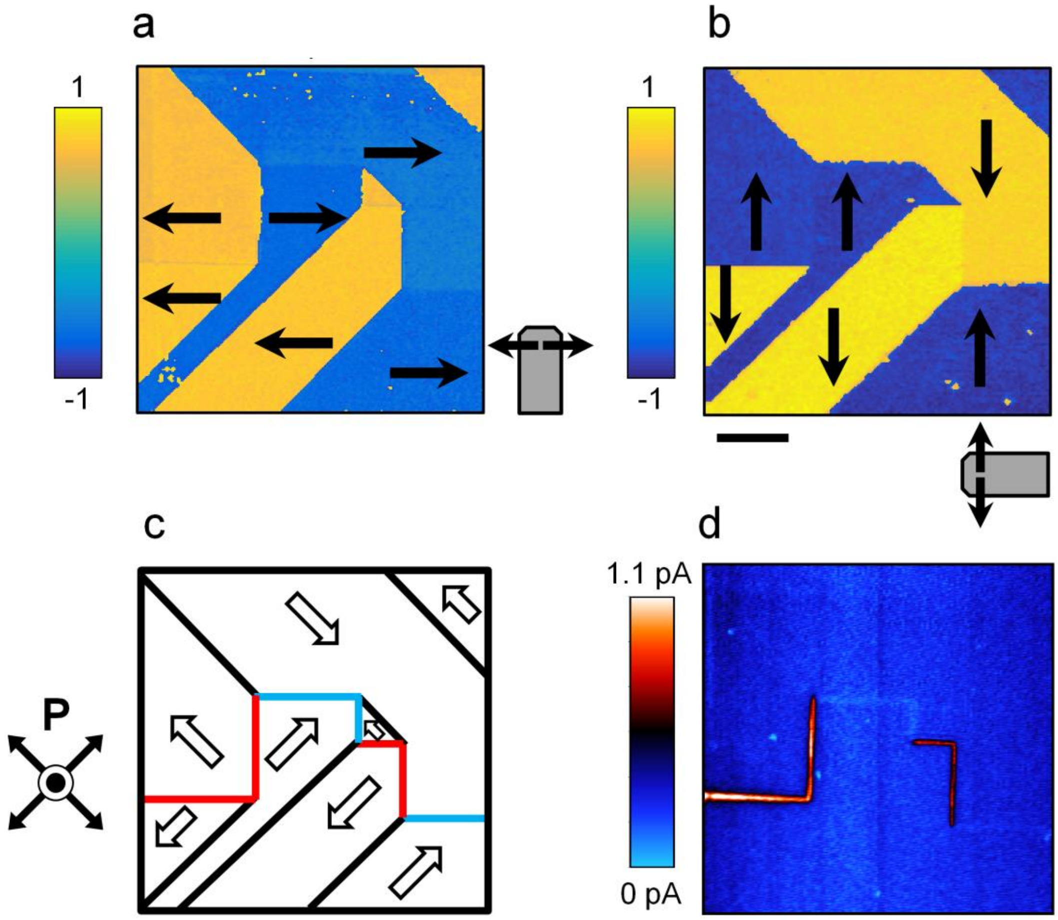

Supplementary Figure 4. Corrected piezoresponse force microscopy and current mapping. (a),(b) Corrected lateral-piezoresponse (PR) maps obtained in 0˚ and 90˚ orientations (rotation of the sample with respect to the cantilever). Black arrows denote sensed polarisation component by lateral piezoresponse force microscopy. (c) Calculated orientation of the polarisation (**P**) in the respective domains. The calculated domain orientations are consistent with both the known domain-wall types in the boracite system and the charged wall profile inferred from the measured current map pattern, (d), obtained with -12 V_{dc} applied to the tip. The scale bar measures 2 μm.

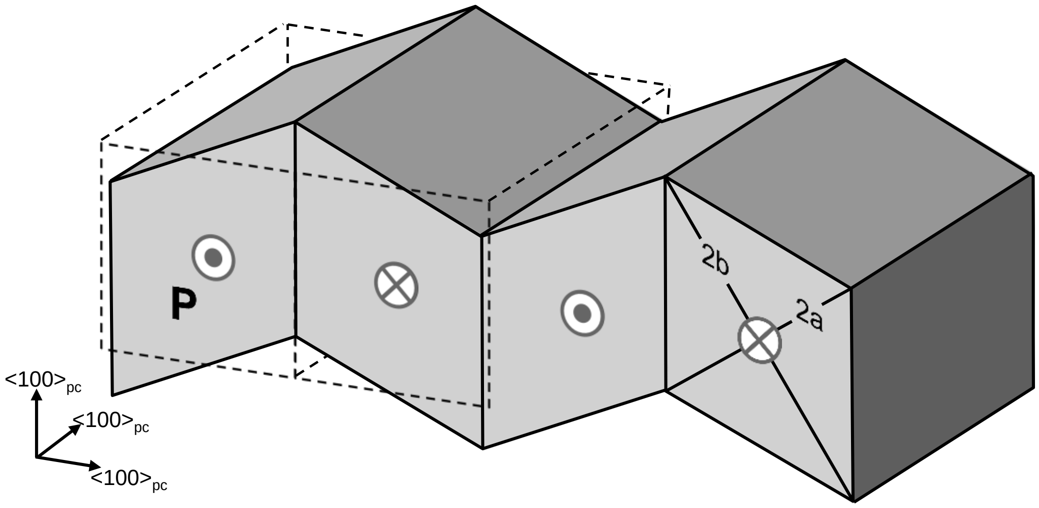

Supplementary Figure 5. Schematic domain structure for uncharged 180º polar boundary. Schematic showing how ferroelastic domains with antiparallel polarisation form mechanically compatible boundaries along $\{100\}_{\text{pseudocubic (pc)}}$ boundaries. The dashed box outline shows schematically the high-temperature cubic structure. Labels *a* and *b* correspond to the orthorhombic axes lengths in the low-temperature ferroelectric phase. 180˚ domains with polarisation (**P**) oriented in the plane of the surface are expected to show a characteristic surface corrugation that is seen experimentally in our local topography mapping, such as in main text Fig. 1d.

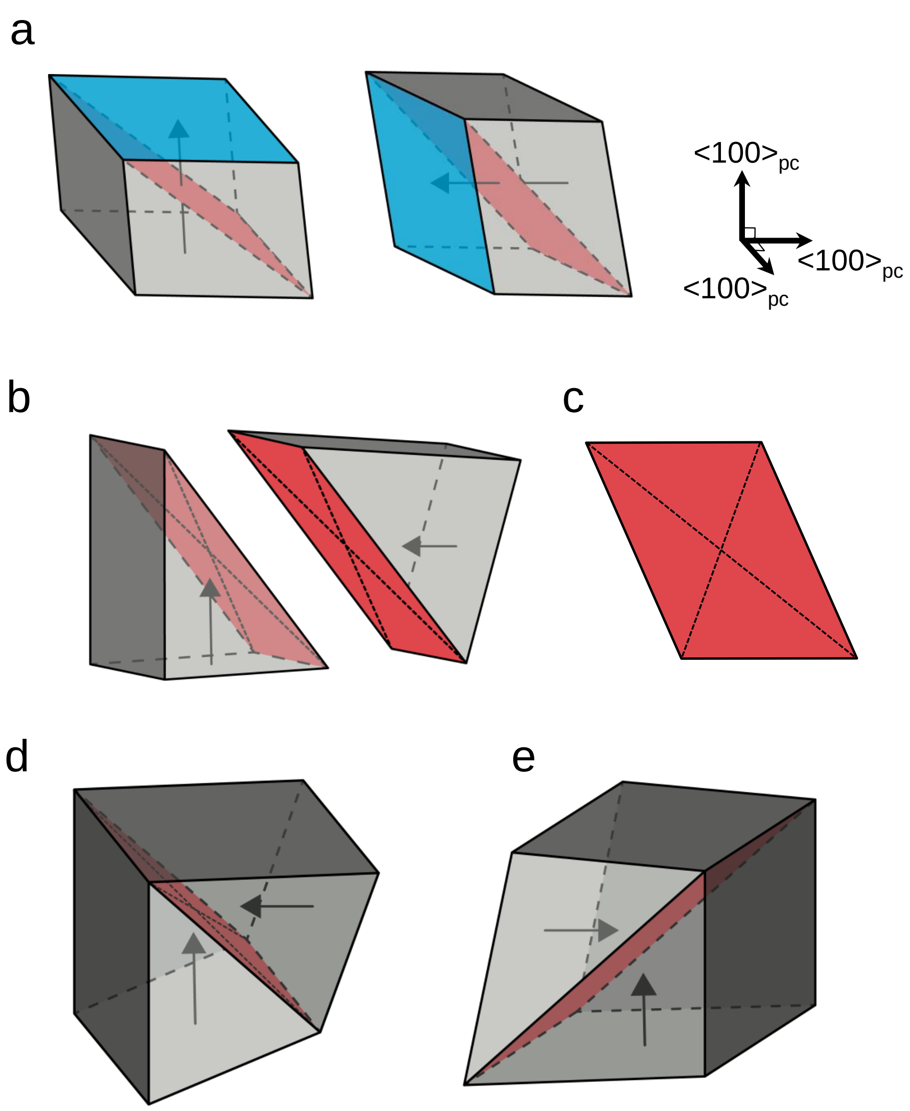

Supplementary Figure 6. Schematic domain structure of charged 90º boundary. (a) Schematic of two orthorhombic phase boracite unit cells with polarisation oriented at a relative angle of 90˚. Blue colouring indicates the sheared face (*ab*-plane of orthorhombic cell) and black arrows denote polarisation direction. The $\{110\}_{pseudocubic\ (pc)}$-type plane is shaded in red. (b) The same sheared unit cells are shown with a section cut along $\{110\}_{pc}$ revealing matching rhomb-like sections, shaded in red, as described by Schmid [4]. The $\{110\}_{pc}$-faced rhomb-like section is shown in (c). The dashed lines denote the long and short-axes; note that they intersect not quite at 90˚. (d) The two unit-cell sections are brought together and rotated such that the rhomb-like sections overlay exactly (the long axes of each rhomb-like section meet and similarly for the short-axes). The necessary relative rotation of the cells gives rise to a depression which converges along the $\{110\}_{pc}$ plane where the cells are joined. Viewed from the rear, an extrusion is observed along $\{110\}_{pc}$ plane (e). This behaviour is seen in our topography maps of ferroelastic $\langle 110\rangle_{pc}$ oriented boundaries.

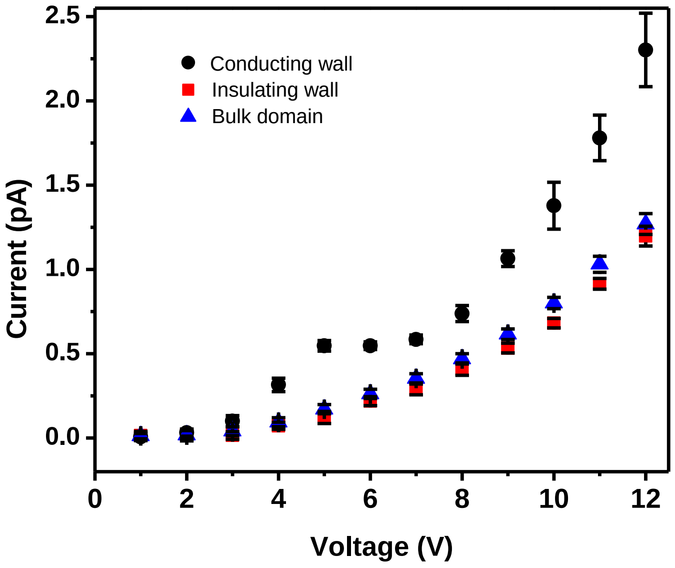

Supplementary Figure 7. Local current-voltage mapping of domain walls. Room temperature current-voltage profile for charged conducting walls (black circles), charged insulating walls (red circles) and bulk domains (blue triangles) measured locally using a biased atomic force microscope probe. Each data point represents average value for measured wall current taken across multiple scan lines at a given applied bias with error bars representing standard deviation of the measured values. On the whole, as tip-bias is increased the discrepancy between the bulk signal and that of conducting walls becomes more pronounced while the difference between insulating walls and the bulk remains subtle. An unexpected plateau feature is observed in the profile of the conducting wall which at present we are unable to rationalise but may be related to the tip-surface contact (although a similar effect is not obvious in the bulk or insulating-wall profiles).

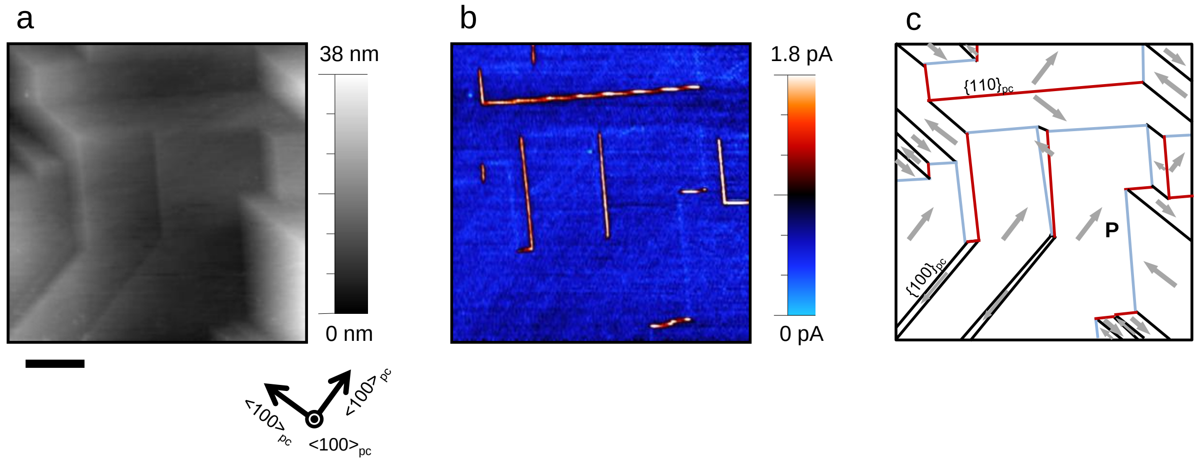

Supplementary Figure 8. Local current mapping and polar structure determined using domain topography. (a) Measured atomic force microscopy topography of a $\{100\}_{pc}$ faced Cu-Cl boracite crystal plate. (b) Spatially resolved current mapping for the area presented in (a). (c) Polar domain map prediction for the same area with grey arrows denoting polarisation direction (**P**). Blue boundaries are head-to-head charged 90° walls, red boundaries are tail-to-tail charged 90° walls, and black boundaries are uncharged 180° walls. The scale bar measures 4 μm.

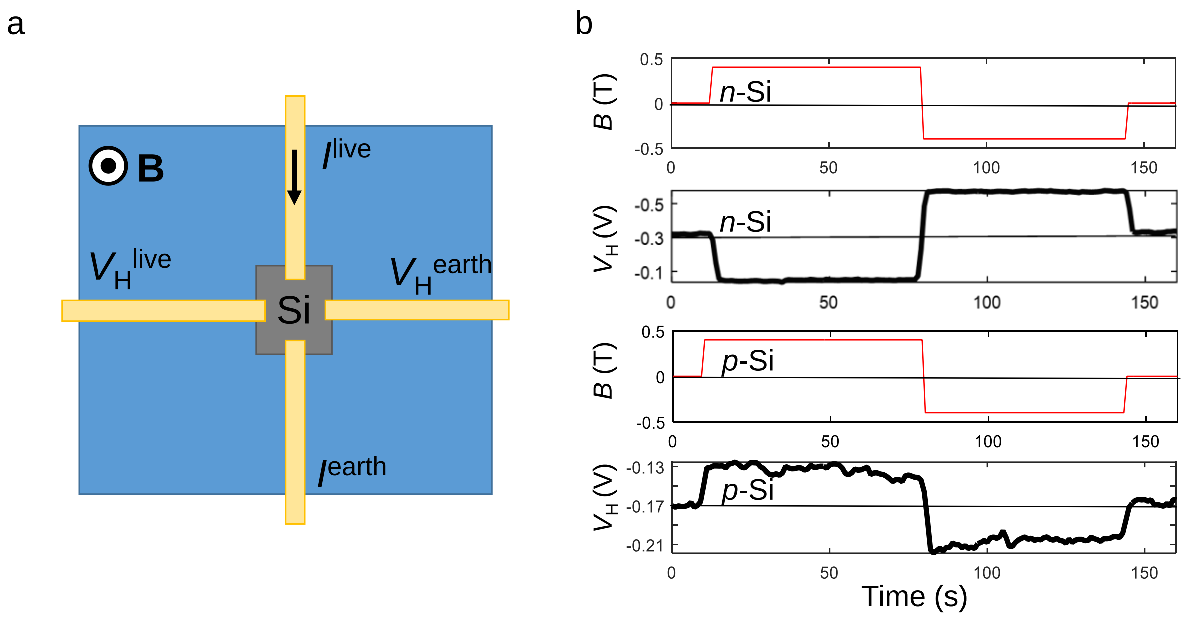

Supplementary Figure 9. Testing Hall effect geometry using doped silicon. (a) Schematic of Hall voltage measurement on doped silicon. The direction of the drive current (*I*) and positive magnetic field (**B**) are indicated and contacts are deposited along the axis where the Hall voltage (V_H) is expected to develop (b) Observed Hall voltage trends for *n*-type and *p*-type silicon samples for time varying magnetic field. Changing the sense of the magnetic field reverses the sign of the Hall voltage. A Hall voltage of the opposite sense is seen to develop for the *n*- and *p*-type samples studied and is consistent with expectations from vector analysis of the Hall Effect equation.

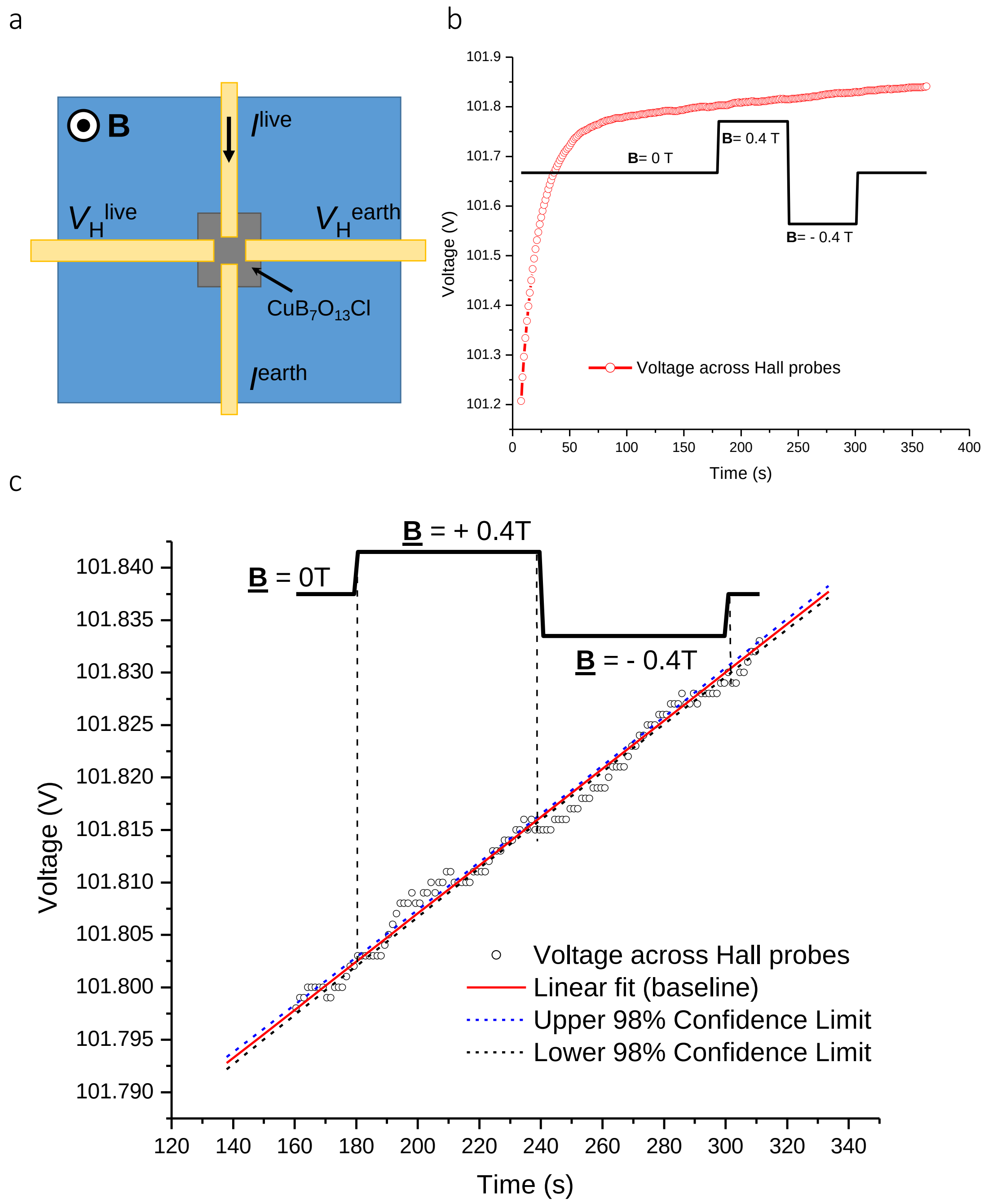

Supplementary Figure 10. Hall effect measurements on Cu-Cl boracite crystal. (a) Schematic of Hall voltage measurement on Cu-Cl boracite crystal. (b) The voltage measured across the Hall probes with 500 nA driven across the sample planar electrodes. (c) The voltage recorded across the contacts oriented perpendicular to the current flow during the application of the magnetic field. Positive field appears to create a positive jump superimposed on the measured transverse voltage and is attributed to the Hall effect. Reversing the sense of the magnetic field leads to a flip in the sense of the developed Hall voltage signal. In general, the observed trend of the voltage jumps agrees with that of *p*-type silicon.

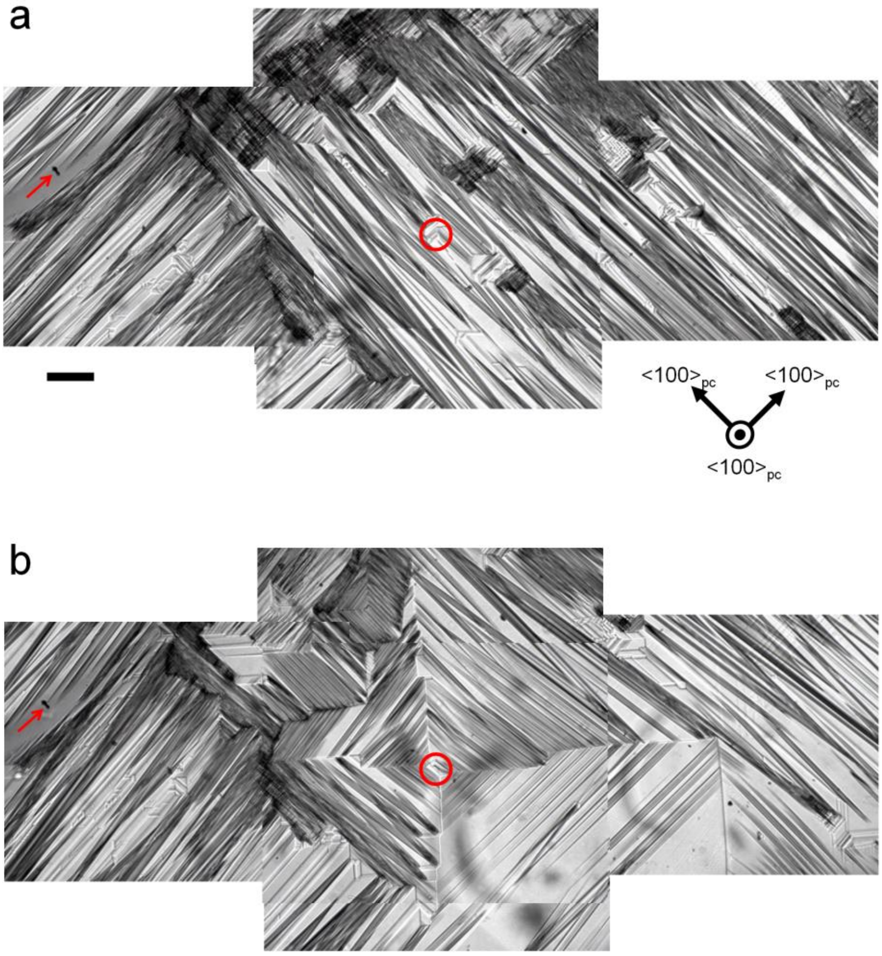

Supplementary Figure 11. Optical microscopy of stress-written conducting domain walls. (a) Montage of transmission optical microscopy images showing (a) a spontaneously forming domain pattern in Cu-Cl boracite after cooling through the phase transition and (b) resulting domain pattern after probe-applied pressure (at 90 ºC) in the region marked with a red circle in (a). Red arrow denotes topographical marker reference. The scale bar measures 200 μm.

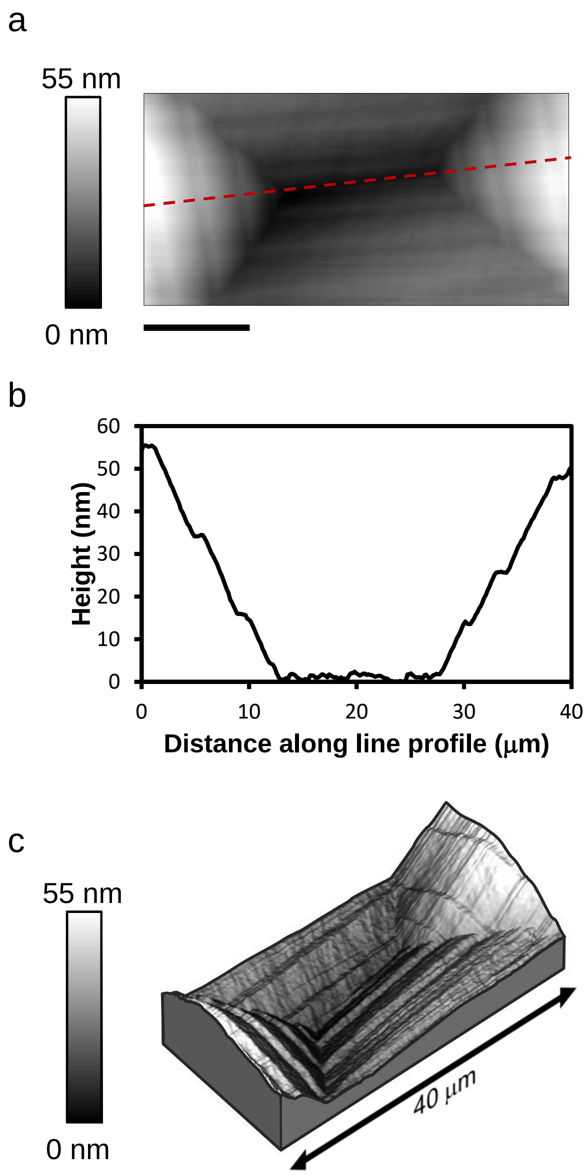

Supplementary Figure 12. Topography depression associated with stress-written domains. (a) Atomic force microscope measured topography surrounding the region where probe-stress is applied. Corrugations in topography surrounding the centre region indicate formation of ferroelastic domains in response to stress (rather than plastic deformation). (b) Line section of topography measured along red-dashed line region in (a). (c) 3d render of topography map shown in (a) shows how the surrounding domain structure generates the sloped topography in response to the applied compressive stress. The scale bar measures 10 μm.

Supplementary Note 1. Interpretation of PFM maps in Cu-Cl boracite: An improper ferroelectric with small spontaneous polarisation

Lateral-piezoresponse force microscopy (L-PFM) routinely provides an excellent means to identify polar domain variants that are completely oriented in the plane of a crystal or thin-film [5]. For proper ferroelectrics, which generally exhibit large spontaneous polarisation (linked to large piezoelectric coefficients), the technique works in a straightforward manner for interpretation of polar variants. However, interpreting piezoresponse signals in ferroelectric systems with small polarisation, such as in some improper ferroelectrics, can pose a challenge due to weak piezoelectric responses. Confounding factors that depend on the material and/or instrumental setup can lead to erroneous results, due to e.g., off-axis coefficients contributing to lateral signal at large a.c. voltages, flexure dominating the actual signal and topographic crosstalk. In the following, we explore how such factors may be accounted for and erroneous PFM measurements corrected in Cu-Cl boracite, a material with spontaneous polarisation an order of magnitude smaller than $BaTiO_3$.

Here we show lateral PFM images ($R\sin\varphi$ maps, where R is measured amplitude of piezoresponse and φ is the phase angle) collected on a single crystal Cu-Cl boracite sample with the tip sample orientations as shown in Supplementary Fig. 2.

Superficially, inspection of the calculated polarisation directions determined from the as-measured L-PFM maps (Supplementary Fig. 2d) suggests that *all* the walls between the domains should exhibit polar discontinuities (some head-to-head and others tail-to-tail). However, this interpretation is erroneous for two key reasons. Firstly, the current maps obtained on this area (Fig. 2a, main text) strongly indicate that *only* the horizontally and vertically aligned walls in Supplementary Fig. 2 show current anomalies (either currents greater than or less than the bulk) and therefore only these walls are likely to be charged. Further to this, the prediction of 180° head-head/tail-tail charged walls (boundaries oriented at 45° to panel edge in Supplementary Fig. 2d) is not consistent with the known domain-wall types in the boracite system. This issue is repeatedly observed across several data sets and suggests that an additional unaccounted-for signal is present in the as-measured piezoresponse maps that precludes straightforward vector-PFM analysis. We attempted study of the sample using high-frequency a.c. methods on our Veeco system as well as on an Asylum Cypher™ system in University College Dublin (in the lab of Dr. B. Rodriguez) to

see if the behaviour was specific only to measurements performed on our instrument at low-frequency but found qualitatively similar results using the different setups.

In boracites, the domain polarisation is known to be totally coupled with the shear distortion that develops through the phase transition (it is a proper ferroelastic and improper ferroelectric) and we therefore consider the possibility that the erroneous signal may be related to the surface shearing detected in topography maps. A cursory look at the derivative of the topography (usually termed as deflection) taken along rows ($\partial z/\partial x$) and columns ($\partial z/\partial y$) as shown in Supplementary Fig. 2e,f reveals a contrast almost identical to the domain maps observed in Supplementary Fig. 2b,c for the two orientations. Such strong correlation observed between topography and PFM phase maps is often an indicator for topographic crosstalk dominating the signal. While some degree of correlation between topography and polar maps may reasonably be expected in these materials (due to the explicit shear strain/polarisation relationship that exists) we nonetheless consider unaccounted-for topography-related signals as the most likely cause for the erroneous PFM maps.

We find strong evidence supporting this view when comparing phase maps for forward/backward scans when weak amplitude signals are recorded (as is often the case), with an example case shown in Supplementary Fig. 3. Here, opposite phase measured by the tip travelling the same scanline on the return path clearly reveals a deflection related contribution that is significant enough to reverse the expected domain-specific phase contrast. While L-PFM signal for a given relative orientation between sample and cantilever should be defined purely by the piezoelectric coefficients of the material and the ferroelectric domain structure, the deflection contribution instead depends on the topography of the sample and the scan direction/path. In such a scenario, the as-measured PFM is most likely to therefore be a vector combination of these two contributions with dependence on the scan orientations.

The sloped domain surfaces measured on the boracite crystal surface extend over heights of only tens of nanometres and it is therefore difficult to attribute the deflection contribution to direct topographic crosstalk. Instead, one possibility is that small mis-orientations/mis-cuts can activate elements of the piezoelectric tensor which would otherwise have not contributed in perfectly oriented crystal surfaces. Using TEM analysis, we have confirmed that the crystal is well oriented but small mis-cuts cannot be fully ruled out. Another possibility is that since we are using large ac voltages (of the order of 5 V_{ac}) to evaluate domain structures even these weak coefficients may contribute significantly if overall signals are low. The situation is

generally avoided in PFM interpretation because of the larger values of polarisation being studied in typical samples.

While it remains a challenge to precisely pinpoint the origin of the deflection type contribution associated with the topography, it may still be possible to extract meaningful polar maps by considering simple ways in which the deflection signal and true piezoresponse may combine and analysing the output using established knowledge of what domain structures are crystallographically possible in the boracite system. The Supplementary Fig. 3 data suggests that the deflection contribution is significant and therefore it may be reasonable to assign comparable weighting to the signals generated from the two contributions. In addition, since L-PFM is sensitive to torsional cantilever displacements we anticipate the relevant topography gradients to be those directed perpendicular to the fast scan axis. Under these assumptions, we find that the PFM response in 0° and 90° orientations can then be corrected by considering various possible linear combinations of the two contributions.

All four possible combinations of the two contributions have been considered (i.e. normalised $\partial z/\partial x$ and $\partial z/\partial y$ added or subtracted to each PFM image) with only one combination generating a domain pattern with allowed domain-wall types that is also consistent with the charged wall picture inferred from our current mapping. These corrected PFM images for the two scan orientations are shown in Supplementary Fig. 4 and are completely consistent with the polar map predicted using solely the ferroelastic domain shearing revealed by the surface domain topography in conjunction with the measured domain-wall orientations. Furthermore, the correction also goes some length to equalise the amplitude contrast across the four domain variants seen in both images (as may be expected since the four in-plane polarisation directions share the same magnitude component perpendicular to the cantilever axis). In summary, the overall PFM response in the improper ferroelectric boracite crystal, with weak spontaneous polarisation, has significant contributions consistent with the topography derivative which can be corrected to achieve a sensible interpretation of the in-plane ferroelectric domain structure.

Supplementary Note 2. Determining domain polarisation from ferroelastic domain structure

In a previous study [6], Zimmermann *et al*. have established the relationship between the form of the structural shearing and the orientation of polarisation developed in the boracite unit cell. They have identified that domain-related surface distortions, such as seen in our topographical maps, can only be associated with polarisation developed in-the-plane of the surface and oriented perpendicular to the gradient vector of the slope associated with the shear deformation. Furthermore, they note that the only strain-free boundaries oriented along $\{100\}_{pc}$ separating in-plane polarised domains are uncharged 180° ferroelectric walls. The next key piece of information is that the only strain-free boundary with composition plane $\{110\}_{pc}$ corresponds to the unusual case of either a head-to-head or a tail-to-tail charged 90º domain wall. Zimmermann and co-workers [6] point out that there is no elastically compatible domain pair that generates an uncharged 90º polar wall (head-to-tail) along this plane. Using this scheme, a consistent set of polar vectors can be assigned for the domains based on the atomic force microscope (AFM) measured topography of the domain walls and predictions for the resulting charge state of each boundary can therefore be obtained. In the following, we apply this polarisation analysis to a different set of domains than in the main text as further supporting evidence that anomalies in the spatially-resolved current map can be successfully rationalised using this charged wall analysis.

A map of AFM measured topography, from a different chemically mechanically polished Cu-Cl crystal plate than that shown in Fig. 1 and 2 in the main text (but from the same $<100>_{pc}$ faced parent single crystal), is presented in Supplementary Fig. 8a. Spatially resolved current mapping of the same area is shown in Supplementary Fig. 8b, showing walls of enhanced conductivity and walls of suppressed conductivity compared to bulk. Using the topographical trace of the ferroelastic domain boundaries in Supplementary Fig. 8a, a prediction for the polar domain map can be generated, using the assumptions that $<100>_{pc}$ boundaries are uncharged 180° boundaries between in-plane polarised domains and $<110>_{pc}$ boundaries are charged 90° boundaries. As can be seen by visual comparison of Supplementary Fig. 8b,c there is a direct correlation between the predicted charge state of the boundary and the measured conductivity anomalies, just as in Fig. 2 of the main text, again supporting the interpretation that polar discontinuities are responsible for the observed transport anomalies. We note that when using the topographic analysis to determine the polar maps, an

interpretation with all of the polar vectors rotated by 180º is also valid (thereby necessitating the use of PFM for absolute determination of polarisation direction).

Supplementary Note 3. Identification of carrier type in Cu-Cl boracite: Hall effect measurements

Direct determination of the carrier type in materials can be achieved by undertaking Hall voltage measurements. The technique has been extensively used to evaluate carrier type and mobilities in semiconductor samples [7]. Here, we employ Hall voltage measurements to shed light on the dominant carrier type in single-crystal Cu-Cl boracite.

The general setup used for the Hall measurements is shown in Supplementary Fig. 9a. The direction of the positive applied magnetic field and current flow, defined by deposited contact pads, are shown in the schematic. To initially test the setup, *n*-type silicon and *p*-type silicon wafer chips are subjected to a time-varying magnetic field and the voltage developed across perpendicular pads is recorded. Offset voltages are measured for both samples. The voltage trends observed for the *n*-type sample are precisely opposite to that of the *p*-type silicon for the same sequence of positive and negative magnetic fields, as expected from vector analysis of the Hall equation. Since all other parameters are kept constant, the change in sign of Hall voltage between the two samples can be easily explained by the different majority carrier types. This simple experiment serves as preparation for investigation of single crystal Cu-Cl boracite where carrier type can be found by comparing the nature of the Hall voltage developed with that obtained for *n*-type or *p*-type silicon.

Following this, the silicon is replaced with a single crystal of Cu-Cl boracite with electrodes deposited as shown in Supplementary Fig. 10a. The resistivity of the crystal is much higher than doped silicon, resistivities ranging from 10^7-10^{11} Ωm have been reported [8], requiring bias application of ~200 V_{dc} to drive 500nA (using a Keithley model 237 source/measure unit). As a result, a significant offset voltage is seen to develop across the Hall contacts (due to electrode misalignment), as shown in Supplementary Fig. 10b. The large resistance of the configuration introduces a delay in development of the offset across the contacts (due to large time constants). We allow the signal to relax for 3 min before a sequence of positive and negative magnetic fields is applied and the resulting trend in Hall voltage observed (Supplementary Fig. 10).

The Hall voltage generated by application of the fields is much smaller than observed for the silicon samples. A zoomed-in view of the voltage recorded across probes during the application of the magnetic field is shown in Supplementary Fig. 10c. Positive field appears to create a positive jump over the baseline (by a few mV). Changing the sense of the magnetic field reverses the sense of the Hall voltage. Overall, the observed trend of the voltage agrees qualitatively with that of *p*-type silicon (although the magnitude of the response and signal-to-noise is much lower). The data shown here therefore suggests that the dominant carrier type in Cu-Cl boracite is also *p*-type in nature.

References

[1] Ito, T., Morimoto, N. & Sadanaga. R. The crystal structure of boracite. *Acta Cryst.* **4**, 310-316 (1951).

[2] Thornley, F. R., Nelmes, R. J. & Kennedy, N. S. J. Structural studies of Cu-Cl-boracite. *Ferroelectrics* **13**, 357-359 (1976).

[3] Torre, L. P., Abrahams, S. C. & Barns. R. L. Ferroelectric and ferroelastic properties of Mg-Cl-boracite. *Ferroelectrics* **4**, 291-297 (1972).

[4] Schmid, H. In: Growth of Crystals (eds Shubnikova, A. V. & Sheftal N. N.) Vol. 7, pp 25-52 (Consultants Bureau, London, 1969).

[5] Ruediger, A. In: Scanning Probe Microscopy of Functional Materials (eds Kalinin S. V. & Gruverman, A.) Ch. 13, 385-405 (Springer, New York, 2011).

[6] Zimmermann, A., Bollmann, W. & Schmid, H. Observations of ferroelectric domains in boracites. *Phys. Status Solidi (a)* **3,**707-720 (1970).

[7] Sze, S. M. & Ng, K. K. *Physics of Semiconductor Devices* 2nd edn 33 (Wiley, New York, 2007).

[8] Schmid, H. & Pétermann, L. A. Dielectric constant and electric resistivity of copper chlorine boracite, $Cu_3B_7O_{13}Cl$ (Cu-Cl-B). *Phys. Status Solidi (a)* **41,** K147-K150 (1977).
